# Vaccine Dispensing Through Private Community Pharmacies in Costa Rica: A National Descriptive Analysis

**DOI:** 10.3390/pharmacy14070146

**Published:** 2026-10-01

**Authors:** Jeaustin Mora-Jiménez, Kevin Cruz-Mora, Sofia Segura-Cano, Yajaira Quesada-Rojas, Jose Gatgens-Gomez, Sebastian Arguedas-Chacón, Esteban Zavaleta-Monestel

**Affiliations:** 1 Health Research Department, Clínica Bíblica, San José 1307-1000, Costa Ricasarguedas@clinicabiblica.com (S.A.-C.); 2 Pharmacy Department, Clínica Bíblica, San José 1307-1000, Costa Rica; kcruzm@clinicabiblica.com; 3 School of Medicine, Universidad de Costa Rica, San José 11501-2060, Costa Rica; sofiaelena.segura@ucr.ac.cr; 4 Unit for the Standardization of Professional Practice, Colegio de Farmacéuticos de Costa Rica, San José 1307-1000, Costa Rica; quesadarojas@gmail.com; 5 Presidency Office, Central American and Caribbean Federation of Pharmacy, San José 1307-1000, Costa Rica; jgatgens360@gmail.com

**Keywords:** community pharmacy services, Costa Rica, yellow fever, human papillomavirus vaccines, influenza vaccines

## Abstract

Background: Pharmacist involvement in immunization has expanded as a strategy to improve access to vaccines. In Costa Rica, private community pharmacies operate under a regulatory framework for vaccination services, yet evidence on private-market vaccine dispensing remains limited. The aim of this study was to characterize vaccine dispensing through private community pharmacies in Costa Rica by dispensed doses, economic value, vaccine category, and provincial distribution, comparing MAT February 2025 and MAT February 2026. Methods: A descriptive, retrospective, national study was conducted using an aggregated secondary IQVIA database of the Costa Rican private pharmaceutical market. Dispensed doses, economic value in USD, percentage variation, and share by vaccine category and province were analyzed; no individual patient, professional, establishment, or nominal vaccination records were used. Results: Dispensed doses increased from 162,664 to 217,387 (+33.6%), while economic value increased from USD 8,700,809 to USD 13,416,717 (+54.2%). Influenza accounted for the highest dispensing volume in MAT February 2026 (87,064 doses). Yellow fever showed the largest between-period increase, from 22,281 to 70,133 doses and from USD 2.7 million to USD 7.0 million. HPV ranked second in economic value (USD 3.6 million). San José accounted for 63.0% of dispensed doses and 52.9% of national private-market economic value. Conclusions: Private-market vaccine dispensing through Costa Rican community pharmacies was higher in MAT February 2026 than in MAT February 2025 in both dispensed doses and economic value, driven primarily by yellow fever. Influenza accounted for the highest dispensing volume, while HPV retained substantial economic weight. These findings characterize vaccine dispensing through the private community pharmacy market but do not establish vaccine administration, vaccination coverage, or clinical or epidemiological outcomes.

## 1. Introduction

Vaccination is one of the public health strategies with the greatest impact and best cost-effectiveness. Its contribution to reducing mortality and gaining healthy life-years exceeds that of many other health interventions [1]. Since 1974, immunization programs have prevented approximately 154 million deaths worldwide, mainly in children under five years of age, who account for about 95% of these lives saved. This impact translates into roughly 9.0 billion life-years gained and 10.2 billion healthy life-years preserved when the reduction in morbidity associated with vaccine-preventable diseases is also considered [1,2].

The role of the pharmacist in immunization programs has progressively expanded internationally, consolidating as a strategy to improve access to vaccination and increase vaccine uptake in different populations. Available evidence shows that pharmacist participation can significantly increase vaccination rates, both when the pharmacist directly administers vaccines and when participating in promotion, education and vaccine recommendation activities. In systematic reviews, the pharmacist acting as an immunizer has been associated with an increase in vaccination rates, with a relative risk of 1.14, whereas their role as a promoter or advocate of vaccination has been linked to an even greater effect, with a relative risk of 1.31 [3]. Likewise, a meta-analysis reported that pharmacist-led immunization programs have a relevant overall impact, with variations by vaccine type, including influenza, herpes zoster and other vaccines [4]. Taken together, this evidence supports the broader role of community pharmacies as complementary access points for vaccination within immunization systems [3,4].

In Costa Rica, immunization operates within a universal healthcare system in which the Caja Costarricense de Seguro Social (CCSS) serves as the public provider of comprehensive healthcare, while the Ministry of Health retains regulatory and stewardship functions [5]. The public healthcare system is financed through pooled social insurance and government contributions, and national resources constitute the predominant source of routine immunization financing [6]. Within this context, pharmacist participation in immunization services is supported by a regulatory and professional framework composed of several complementary provisions.

Vaccines included in Costa Rica’s official immunization schedule are provided free of charge through the public healthcare system to the populations for whom they are indicated. Although the national immunization program provides broad population access, eligibility and prioritization for specific vaccines or seasonal campaigns may vary according to age, clinical risk, pregnancy status, occupation, or other predefined criteria. For example, the 2025 seasonal influenza campaign initially prioritized defined age and risk groups, whereas HPV vaccination is incorporated into the national age-based vaccination schedule [7].

Private vaccination services operate alongside this publicly funded program and are available to individuals who elect to receive vaccines through the private sector. Private use may reflect factors such as convenience, preference for private healthcare, timing or accessibility of vaccination, or preference for a particular vaccine product. However, the present IQVIA dataset does not capture the reasons underlying individual private-market purchases. Therefore, vaccine dispensing through private community pharmacies should not be interpreted as evidence of unmet public-sector need, restricted access, or public-sector supply limitations unless such circumstances are independently documented.

The National Vaccination Law, Law 8111, regulates the selection, procurement and availability of vaccines in the country, as well as the role of the National Vaccination and Epidemiology Commission in defining the official list of vaccines [8]. Complementarily, the National Vaccination Standard, Decree 37808-S, establishes the technical criteria for the indication, contraindication, application, recording, storage and disposal of vaccines, applicable to authorized public and private services [7]. Likewise, the Manual of Standards for the Licensing of Pharmacies, Decree 31969-S, incorporates requirements for the storage of products requiring a cold chain and for the provision of vaccination services [7].

In this context, the 2014 COLFAR Protocol for injectables and vaccination services in private pharmacies defines requirements for human resources, physical facilities, documentation and procedures for the administration of injectables and vaccines, while the professional guideline COLFAR G-PP-287 specifically operationalizes Pharmaceutical Immunization Services from the community pharmacy, aimed at permanent or substitute regent pharmacists who participate in or wish to implement these services [9].

Previous studies in Costa Rica have characterized immunization services in private community pharmacies primarily through pharmacist-reported surveys, focusing on professional practices, service availability, and the implementation of vaccination-related activities. Regional studies in Central America have similarly examined community pharmacy services from the perspective of pharmacy professionals. However, these approaches do not objectively quantify the volume of vaccines dispensed through the private community pharmacy market, their economic value, their distribution across vaccine categories, or their geographic distribution. Consequently, an important knowledge gap remains regarding the scale and composition of vaccine dispensing through the private community pharmacy sector in Costa Rica. To our knowledge, this is the first national market-based analysis in Costa Rica to characterize private-community-pharmacy vaccine dispensing using aggregated pharmaceutical market data. Accordingly, the present study addressed the following research question: How did vaccine dispensing through private community pharmacies in Costa Rica differ between MAT February 2025 and MAT February 2026 in terms of dispensed doses, economic value, vaccine category, and provincial distribution? Therefore, the objective of this study was to characterize these dispensing patterns using aggregated private-market data for Costa Rica.

## 2. Materials and Methods

### 2.1. Study Design

A descriptive, retrospective, national study was conducted using an aggregated secondary database of vaccine dispensing activity in the private market of Costa Rica. The analysis focused on preventive vaccines dispensed in private community pharmacies during the MAT February 2025 and MAT February 2026 periods. The study was reported in accordance with the STROBE statement, and the completed checklist is provided as Supplementary File S1.

### 2.2. Data Source

The information source was a secondary database of the private pharmaceutical market obtained from IQVIA data for Costa Rica. The dataset reflected sell-out activity from private community pharmacies to the final consumer; therefore, the reported units represent vaccine doses dispensed through the private community pharmacy channel rather than distributor-to-pharmacy sell-in. The source dataset included product-level information on commercial presentation, manufacturer, therapeutic classification within the J07 vaccine group, vaccine category, province, dispensed units, and economic value expressed in US dollars. Geographic analyses were based on the province reported in the IQVIA dataset for each record.

For this analysis, a cleaned sheet of the original database was used, retaining only preventive vaccines and excluding immunological products that did not correspond to preventive vaccination in the community pharmacy context. The source dataset was organized at the commercial-product level, with each product record classified according to an ATC-based therapeutic classification within the J07 vaccine group and its corresponding subgroups. For the present analysis, the therapeutic classification provided in the source dataset, together with the antigen composition of each vaccine, was used to assign each eligible record to a single mutually exclusive analytical vaccine category. Commercial product names were used only during the record-review and classification stage and were not used as analytical categories. Products corresponding to the same vaccine type were aggregated into a common analytical category irrespective of commercial brand. Combination vaccines were retained as distinct analytical categories according to their antigen composition (e.g., Tdap, DTaP-polio, hexavalent, and MMR) and were not apportioned among their individual antigen components, thereby avoiding double counting. The ATC-based therapeutic classification and analytical vaccine-category mapping used in the study are provided in Supplementary Table S1.

Although product-level classification and presentation information was available in the source dataset, the data were aggregated by vaccine category and province for the main descriptive analysis. Commercial product names, manufacturers, presentations, and source-level product identifiers are not reproduced in the Supplementary Materials because the underlying IQVIA dataset was obtained under a commercial license and is not publicly available.

The analytical dataset available to the investigators did not include information on the number or proportion of individual private pharmacies contributing to the underlying IQVIA data, the proportion of private vaccine sales directly captured, or the sampling, weighting, and projection parameters used in the generation of the database. These coverage parameters could therefore not be independently quantified from the available data. Accordingly, the term “national” in this study refers to the geographic scope of the analysis, which included data reported across all seven provinces of Costa Rica, and does not imply complete capture of all private community pharmacies or all private vaccine dispensing in the country. In addition, the database did not include corresponding public-sector vaccine dispensing or administration data; consequently, the proportion of total vaccine utilization in Costa Rica represented by the private-market data could not be estimated.

### 2.3. Analysis Period

The analysis used MAT February 2025 and MAT February 2026, which were the consecutive 12-month MAT periods available in the IQVIA dataset obtained for this study. The February endpoint therefore reflects the reporting period of the available data source rather than an epidemiological, seasonal, or analytical selection criterion. Complete calendar-year datasets for 2024 and 2025 were not available within the dataset provided to the investigators. MAT, from the English “moving annual total,” corresponds to the cumulative total over the 12 months ending in February of each year. Therefore, MAT February 2026 represents the 12-month period ending in February 2026, while MAT February 2025 represents the immediately preceding comparable 12-month period.

MAT data were used rather than isolated monthly observations because each MAT value summarizes dispensing activity over a complete 12-month period and is therefore less sensitive to fluctuations occurring in any single month. However, the use of aggregated MAT values does not permit direct assessment of monthly or seasonal variation.

### 2.4. Unit of Analysis

The unit of analysis consisted of aggregated records of preventive vaccine dispensing in the private market, grouped by vaccine category and province. All vaccine products included in the analytical dataset were recorded as single-unit (×1) commercial presentations; therefore, one dispensed unit corresponded to one vaccine dose dispensed, and no additional unit-to-dose conversion was required. Dispensed doses should not be interpreted as confirmed doses administered or as the number of persons vaccinated. The database does not contain individual vaccination records or the demographic and eligibility information required to calculate vaccine-specific population coverage or administered-dose rates.

### 2.5. Inclusion Criteria

All commercial-product records classified within the J07 vaccine group in the IQVIA source dataset and corresponding to preventive human vaccines during either MAT February 2025 or MAT February 2026 were eligible for inclusion. Eligibility was verified at the record level using the ATC-based therapeutic classification provided in the source dataset together with the vaccine antigen composition. Records corresponding to the same vaccine type were subsequently aggregated into their predefined analytical vaccine category. No eligible preventive vaccine was excluded on the basis of dispensing volume, manufacturer, commercial presentation, or whether dispensing activity was recorded in one or both MAT periods.

Vaccine products were grouped into broad clinical categories, such as influenza, yellow fever, human papillomavirus, Tdap, hepatitis A, hepatitis B, pneumococcal, meningococcal, varicella, and hexavalent vaccines, among others. The complete ATC-based therapeutic classification and analytical category mapping is provided in Supplementary Table S1.

The main figures display the principal vaccine categories for clarity of presentation; therefore, the sum of the categories shown individually in the figures does not necessarily equal the overall dispensing total. Products not displayed separately remained included in the overall analytical totals. The ATC-based therapeutic classification and analytical vaccine-category mapping used in the study are provided in Supplementary Table S1, while complete category-level results are provided in Supplementary Table S2.

### 2.6. Exclusion Criteria

Products within the source dataset that did not correspond to preventive vaccines, such as oral immunostimulants, antivenoms, or other non-vaccine immunological products, were excluded. Empty rows, totals, subtotals, and duplicate records that could alter the aggregated calculations were also excluded. No preventive vaccine product available in the source dataset and meeting the inclusion criteria was excluded on the basis of dispensing volume, commercial presentation, manufacturer, or vaccine category.

### 2.7. Variables Analyzed

The main variables of the study included vaccine doses dispensed (reported as units in the source database) and economic value in US dollars for the MAT February 2025 and MAT February 2026 periods. Economic value corresponded to the aggregate sell-out value associated with vaccine doses dispensed to the final consumer, as reported in the IQVIA dataset. The analytical file was provided with monetary values already expressed in US dollars; therefore, no currency conversion was performed by the investigators. No inflation adjustment was applied, and economic values were analyzed as nominal values as provided in the source dataset.

In addition, the vaccine category and the province of dispensing were considered as grouping variables for the analysis. From these variables, derived indicators were calculated, including the percentage variation between the two MAT periods, the percentage share of each vaccine category within the national total, the provincial percentage share, and the distribution of economic value by category and province. Average economic value per dispensed dose was also calculated by dividing the aggregate economic value by the corresponding number of dispensed doses, both overall and by vaccine category.

### 2.8. Data Analysis

A descriptive analysis was performed using absolute frequencies, percentages, economic values and percentage variations. Dispensed doses and economic values were aggregated at the national level, by vaccine category and by province. The percentage variation between MAT February 2025 and MAT February 2026 was calculated using the following formula:

Percentage variation = [(MAT 2026 value − MAT 2025 value)/MAT 2025 value] × 100 (1)

The percentage share of each vaccine category was calculated by dividing its dispensed doses or economic value by the corresponding national total for the same period. Likewise, the provincial percentage share was calculated by dividing the dispensed doses or economic value of each province by the national total for MAT February 2026. The economic analysis was expressed in US dollars.

To facilitate population-standardized comparisons across provinces, crude private-market vaccine dispensing rates per 1000 inhabitants were calculated for MAT February 2026 by dividing the number of dispensed doses in each province by the corresponding projected provincial population and multiplying by 1000. Provincial population denominators were obtained from the 2025 subnational population estimates of the Instituto Nacional de Estadística y Censos (INEC) of Costa Rica. These rates represent private-market vaccine dispensing relative to the total provincial population and should not be interpreted as vaccination coverage, administered-dose rates, or the proportion of individuals vaccinated.

To further characterize the change in total economic value between MAT February 2025 and MAT February 2026, a descriptive additive decomposition was performed at the vaccine-category level. The total change in economic value was separated into three components: a volume effect, reflecting the change associated with the overall number of dispensed doses while holding the MAT February 2025 market composition and average unit values constant; a mix effect, reflecting changes in the relative distribution of dispensed doses across vaccine categories while holding MAT February 2025 category-specific average unit values constant; and an average unit-value effect, reflecting changes in the average economic value per dispensed dose within vaccine categories. Because the available economic data were aggregated, the latter component was interpreted as an average unit-value effect rather than as a pure transaction-price effect.

No hypothesis tests, inferential models, or multivariate adjustments were performed because the objective of the study was descriptive and the data were available in aggregate form. Sampling-based confidence intervals were not calculated because the analytical dataset did not provide the underlying sampling frame, sampling weights, projection parameters, or variance estimates required to derive statistically valid measures of sampling uncertainty. To assess the influence of the vaccine category showing the largest between-period change on the overall estimates, a sensitivity analysis was performed by recalculating national dispensing volume and economic value after excluding the yellow fever vaccine.

### 2.9. Ethical Considerations

The study used an aggregated secondary database of the private pharmaceutical market, without identifiable individual information on patients, health professionals, prescribers, pharmacies or specific establishments. The database did not include medical records, nominal vaccination records, individual clinical data or variables that would allow direct or indirect identification of individuals. In accordance with the Biomedical Research Regulatory Law of Costa Rica, Law No. 9234, the framework for review by a Scientific Ethics Committee applies to biomedical research involving human beings in health matters; since this study did not involve human participants, clinical intervention, sample collection or access to personal health data, approval by a Scientific Ethics Committee was not considered necessary. Likewise, as it did not process personal or sensitive data, the analysis did not entail risks associated with the processing of information protected under Law No. 8968 on the Protection of Individuals with Regard to the Processing of their Personal Data. The results are interpreted solely as aggregate indicators of dispensing activity and economic value of the private vaccine market, and not as individual clinical data, population vaccination coverage or evaluation of health outcomes.

## 3. Results

During the study period, an increase was observed in the national dispensing activity of preventive vaccines in private community pharmacies in Costa Rica. As shown in Table 1, dispensed doses rose from 162,664 in MAT February 2025 to 217,387 in MAT February 2026, representing an increase of 33.6%. In parallel, the associated economic value increased from USD 8,700,809 to USD 13,416,717, equivalent to an increase of 54.2%. These results show higher dispensed volume and economic value in MAT February 2026 compared with MAT February 2025.

Figure 1 shows the main vaccine categories by dispensed doses. Influenza was the highest-volume category in both periods, increasing from 72,943 doses in MAT February 2025 to 87,064 doses in MAT February 2026. Yellow fever showed the largest between-period increase, rising from 22,281 to 70,133 doses, positioning it as the second-highest-volume category in the 2026 period. HPV also increased, from 22,551 to 26,385 doses. In contrast, some categories showed a reduction in doses, including Tdap, hepatitis A, pneumococcal, meningococcal, and varicella. Overall, the results show that the private market for preventive vaccines was concentrated mainly in influenza, yellow fever and HPV.

The vaccine categories not displayed individually in the main figures accounted collectively for 8270 dispensed doses in MAT February 2025 and 3689 doses in MAT February 2026, corresponding to 5.1% and 1.7% of total dispensing, respectively, and representing a 55.4% decrease between periods. This group comprised MMR, rotavirus, DTaP-polio, herpes zoster, rabies, RSV, and typhoid vaccines. Complete category-level results for both MAT periods, including dispensed doses, market share, economic value, percentage change, and average economic value per dispensed dose, are provided in Supplementary Table S2.

Regarding economic value by vaccine category (Figure 2), yellow fever was the category with the greatest weight in MAT February 2026, increasing from approximately USD 2.7 million in MAT February 2025 to USD 7.0 million in MAT February 2026. HPV ranked second in economic value, with an increase from USD 2.9 million to USD 3.6 million. Although influenza was the category with the highest volume of dispensed doses, its economic contribution was lower compared with yellow fever and HPV, reaching approximately USD 1.2 million in MAT February 2026. Overall, these findings show that the economic value of the private vaccine market was concentrated mainly in yellow fever and HPV.

The percentage variation by vaccine category showed heterogeneous behavior between MAT February 2025 and MAT February 2026 (Figure 3). Yellow fever showed the greatest relative growth, with an increase of 214.8%, well above the rest of the categories. Increases were also observed in influenza, HPV, hepatitis B and the hexavalent vaccine, although of lower magnitude. In contrast, several categories showed a reduction in dispensed doses, especially Tdap, varicella, meningococcal and hepatitis A. Overall, yellow fever showed the largest absolute and relative increase among the vaccine categories analyzed, while several other vaccine categories had lower dispensing volumes in MAT February 2026 than in MAT February 2025.

To assess the influence of yellow fever vaccine dispensing on the overall between-period estimates, a sensitivity analysis was performed excluding this vaccine category. Yellow fever accounted for 47,852 of the 54,723 additional doses observed between MAT February 2025 and MAT February 2026, corresponding to 87.4% of the absolute increase in dispensing volume. It also accounted for USD 4.33 million of the total USD 4.72 million increase in economic value (91.8%). After excluding yellow fever, total vaccine dispensing increased from 140,383 to 147,254 doses (+4.9%), while economic value increased from USD 6.00 million to USD 6.39 million (+6.4%). By comparison, when all vaccine categories were included, dispensing volume increased by 33.6% and economic value by 54.2%.

The provincial distribution (Figure 4) showed a marked concentration of the private market for preventive vaccines in San José during MAT February 2026. This province concentrated 136,883 doses, equivalent to 63.0% of the national total, and USD 7,093,630, corresponding to 52.9% of the total economic value. Alajuela was the second province in share, with 29,270 doses (13.5%) and USD 2,181,103 (16.3%), followed by Heredia, Cartago, Guanacaste, Puntarenas and Limón.

When standardized using the 2025 projected provincial population, San José also showed the highest crude private-market dispensing rate, at 82.4 doses per 1000 inhabitants. The corresponding rates were 28.3 per 1000 in Heredia, 27.5 in Cartago, 27.2 in Alajuela, 23.2 in Guanacaste, 13.7 in Puntarenas, and 9.9 in Limón. These indicators represent population-standardized private-market dispensing and should not be interpreted as vaccination coverage.

Although Limón had the lowest absolute share in dispensed doses and economic value, it showed the largest percentage increase between the two MAT periods in dispensed doses, with an increase of 101.6%. Likewise, Cartago, Guanacaste, and Puntarenas showed increases exceeding 48% between the two MAT periods, indicating higher private-market vaccine dispensing in these provinces in MAT February 2026 than in MAT February 2025.

Comparison of the two MAT periods showed that San José increased from 107,617 dispensed doses in MAT February 2025 to 136,883 in MAT February 2026 (+27.2%), while its share of national dispensing decreased from 66.2% to 63.0%. Alajuela increased from 20,462 to 29,270 doses (+43.0%), Heredia from 11,594 to 15,491 (+33.6%), Cartago from 9717 to 14,669 (+51.0%), Guanacaste from 6359 to 9607 (+51.1%), Puntarenas from 4669 to 6940 (+48.6%), and Limón from 2246 to 4527 (+101.6%). Thus, although San José remained the province with the largest absolute dispensing volume, its relative share decreased as several other provinces showed larger proportional increases.

## 4. Discussion

### 4.1. Changes in Private Vaccine Dispensing Between MAT Periods

Vaccine dispensing through the Costa Rican private community pharmacy market was higher in MAT February 2026 than in MAT February 2025, with dispensed doses increasing by 33.6% and economic value by 54.2%. However, the sensitivity analysis showed that this aggregate change was strongly concentrated in the yellow fever category. After excluding yellow fever, the between-period increase was reduced to 4.9% for dispensed doses and 6.4% for economic value, indicating that the overall market change did not reflect a uniform increase across vaccine categories.

Conversely, several vaccine categories showed lower private-market dispensing in MAT February 2026 than in MAT February 2025. Tdap decreased by 32.8%, varicella by 25.1%, meningococcal vaccines by 19.1%, and hepatitis A by 13.6%. These category-specific decreases further support the interpretation that the aggregate increase in private-market vaccine dispensing was heterogeneous rather than generalized across vaccine categories. Because the available data are aggregated and do not include information on product availability, public-sector supply, eligibility criteria, prescribing patterns, or reasons for purchase, the factors underlying these decreases cannot be determined from the present analysis.

Heterogeneous patterns across vaccine categories have also been reported internationally, although most available studies have evaluated vaccine administration rather than dispensing. In the United States, for example, the number of routine vaccines administered in retail pharmacies increased from 3.4 million in 2015 to 13.3 million in 2023, while analyses of pharmacy-based vaccination have shown marked differences according to vaccine type, with influenza, COVID-19, and herpes zoster accounting for a substantially greater proportion of pharmacy-based vaccination than categories such as HPV [10]. Similar category-specific patterns have been reported in other settings, including Australia, where pharmacy-based vaccination initially concentrated on influenza and COVID-19 before expanding to additional vaccine categories [11].

Although these international data are not directly comparable with the present analysis because they primarily measure vaccine administration, whereas the IQVIA dataset captures dispensing, the comparisons are presented solely to contextualize differences in pharmacy-based vaccine activity across categories and should not be interpreted as evidence of comparable vaccination uptake or coverage in Costa Rica. In the present analysis, this category-specific concentration was particularly evident for yellow fever. Because only two consecutive MAT periods were available, the observed differences should be interpreted as between-period changes rather than evidence of a sustained temporal trend. These findings are also specific to the private pharmaceutical market. Because the IQVIA dataset does not include corresponding vaccine dispensing or administration data from the Costa Rican public healthcare system, the relative contribution of private community pharmacies to total national vaccine utilization cannot be quantified. Consequently, the observed dispensing volumes should not be considered representative of a defined proportion of national vaccination activity.

### 4.2. Dissociation Between Volume and Economic Value

The distribution of dispensing volume differed from that of economic value across vaccine categories. Influenza accounted for the highest number of dispensed doses in MAT February 2026, whereas yellow fever and HPV represented the largest components of economic value. Thus, the vaccine categories with the greatest dispensing volume were not necessarily those contributing the greatest economic value to the private market. This distinction highlights the importance of considering both measures when characterizing vaccine-market composition, as dispensing volume and economic value describe different dimensions of market activity.

### 4.3. Yellow Fever: Between-Period Increase and Epidemiological Context

Yellow fever showed the largest absolute and relative increase among the vaccine categories analyzed. The sensitivity analysis further showed that yellow fever accounted for 87.4% of the absolute increase in dispensed doses and 91.8% of the increase in economic value between MAT February 2025 and MAT February 2026. Therefore, most of the aggregate between-period change observed in the private vaccine market was concentrated in this vaccine category.

Marked changes in yellow fever vaccine utilization have also been documented in other settings during periods of epidemiological or regulatory change [12]. During the 2016–2018 yellow fever outbreak in Brazil, expansion of transmission into previously unaffected areas was accompanied by progressive expansion of vaccination recommendations and emergency strategies to increase vaccine availability [13,14]. Similarly, during the 2016–2018 yellow fever vaccine shortage in the United States, changes in vaccine availability required the implementation of an expanded-access program to maintain vaccination services [13,15]. International vaccination policy has also influenced yellow fever vaccine requirements; for example, the 2016 amendment to the International Health Regulations established lifetime validity of a single documented yellow fever vaccination, replacing the previous 10-year revaccination requirement [16]. Because a single documented yellow fever vaccination has lifetime validity, recurrent demand from previously vaccinated individuals would not be expected to arise from routine revaccination requirements; therefore, the persistence of the marked increase observed in this study cannot be inferred from the two MAT periods analyzed. Together, these experiences illustrate that yellow fever vaccine utilization may be particularly sensitive to changes in epidemiological conditions, supply, and travel-related regulatory requirements [16].

The increase in private-market yellow fever vaccine dispensing observed in Costa Rica occurred within a comparable period of regional epidemiological activity and regulatory change. The Pan American Health Organization reported renewed yellow fever transmission in the Americas during 2025 and continued transmission into 2026 [17,18]. During the same period, the Costa Rican Ministry of Health introduced and subsequently updated yellow fever vaccination requirements for travelers to or from countries considered at risk [19]. Official Ministry of Health communications also documented periods of shortage and limited availability of yellow fever vaccine in the Costa Rican private market during 2025. In response, the Ministry implemented extraordinary public vaccination campaigns to facilitate access for eligible travelers and other prioritized groups. These supply-related circumstances occurred concurrently with the epidemiological and regulatory developments described above and provide additional context for the marked increase in private-market dispensing observed during the study period [20].

However, the IQVIA dataset does not include vaccination indication, travel history, reason for purchase, individual demand characteristics, or contemporaneous public-sector vaccine availability. Consequently, the present study cannot determine whether the observed increase was caused by regional transmission, travel-related requirements, supply constraints, or other factors, nor can it quantify the relative contribution of these factors. Furthermore, the increase in dispensed doses cannot be interpreted as an equivalent increase in doses administered, the number of individuals vaccinated, vaccine uptake, or vaccination coverage. The finding should therefore be interpreted as a marked category-specific increase in private-market dispensing occurring contemporaneously with epidemiological and regulatory changes rather than as evidence of a causal relationship or a sustained structural market trend.

### 4.4. Human Papillomavirus: Dispensing Pattern and Economic Contribution

The HPV vaccine showed a moderate between-period increase, with dispensed doses rising from 22,551 in MAT February 2025 to 26,385 in MAT February 2026, while its economic value increased from approximately USD 2.9 million to USD 3.6 million. Although HPV dispensing volume remained substantially lower than that of influenza, HPV represented one of the vaccine categories with the greatest economic value in the private market. This pattern further illustrates that dispensing volume and economic value do not necessarily follow the same distribution across vaccine categories [21].

International studies based on immunization registries and administrative claims have also shown that HPV vaccination occupies a comparatively smaller share of pharmacy-based vaccination than categories such as influenza or herpes zoster. In a Wisconsin immunization registry analysis, pharmacies accounted for 39% of zoster doses and 20% of influenza doses, compared with only 0.2% of HPV doses [22]. Similarly, Medicaid and commercial claims analyses have shown that although the proportion of HPV vaccination occurring in pharmacies has increased over time, most HPV vaccination continues to occur in non-pharmacy settings [23].

### 4.5. Geographic Distribution of Private-Market Vaccine Dispensing

Private-market vaccine dispensing was geographically concentrated in San José, which remained the province with the largest dispensing volume in both MAT periods. Dispensed doses in San José increased from 107,617 in MAT February 2025 to 136,883 in MAT February 2026 (+27.2%); however, its share of national private-market dispensing decreased from 66.2% to 63.0%. This reduction in relative share occurred because several other provinces experienced faster proportional increases in dispensing. Thus, although San José remained the dominant provincial market, the results suggest a modest relative redistribution of private-market vaccine dispensing toward other provinces between the two MAT periods. Alajuela represented the second-largest provincial share in MAT February 2026, while the remaining provinces contributed smaller proportions.

Population-standardized analysis showed that San José also had the highest crude private-market vaccine dispensing rate, at 82.4 doses per 1000 inhabitants. The corresponding rates were 28.3 per 1000 inhabitants in Heredia, 27.5 in Cartago, 27.2 in Alajuela, 23.2 in Guanacaste, 13.7 in Puntarenas, and 9.9 in Limón. Although these rates facilitate comparison across provinces with different population sizes, they represent private-market dispensing relative to the total provincial population and should not be interpreted as vaccination coverage, administered-dose rates, or the proportion of individuals vaccinated.

This geographic concentration may be considered alongside the territorial distribution of private pharmacies in Costa Rica, where a substantial proportion of pharmacies are located within the Greater Metropolitan Area [24]. However, the present dataset does not include pharmacy density, travel distance, socioeconomic characteristics, individual-level information, or corresponding public-sector vaccination activity by province. Consequently, the observed provincial distribution should be interpreted as a pattern of private-market dispensing rather than as a direct measure of geographic access, unmet need, or vaccination coverage.

These patterns should also be interpreted within Costa Rica’s predominantly public immunization system. Vaccines included in the official national schedule are provided free of charge to the populations for whom they are indicated, while private vaccination services operate alongside the public program. Consequently, higher or lower private-market dispensing in a province cannot be assumed to reflect deficiencies in public vaccine provision. Private use may be influenced by convenience, preference for private care, timing, product choice, eligibility criteria, supply conditions, or other factors that were not captured in the IQVIA dataset.

International geospatial studies have shown that community pharmacies can contribute to the spatial availability of vaccination services, including in rural settings. For example, an analysis from South Carolina found that spatial access to pharmacies for HPV vaccination was comparable between some rural and urban areas, although geographic proximity alone did not ensure vaccine uptake [25]. These international findings provide contextual evidence regarding the potential geographic role of community pharmacies but should not be interpreted as directly transferable to Costa Rica, given differences in healthcare organization, pharmacy regulation, public-sector vaccine provision, and the scope of pharmacy-based vaccination across jurisdictions.

### 4.6. Practice Context for Community Pharmacy Immunization in Costa Rica

Although the present study evaluates vaccine dispensing rather than vaccine administration or specific pharmacist-led interventions, the findings should be interpreted within the regulatory and professional context of community pharmacy practice in Costa Rica. Community pharmacists may administer vaccines in private pharmacies when the service complies with the applicable national regulations and professional guidance. These requirements include appropriate infrastructure, cold-chain management, documentation, professional oversight, and procedures for the safe handling and administration of vaccines and other injectable medicines [9]. The professional guidance issued by the Colegio de Farmacéuticos de Costa Rica specifically describes the requirements and procedures that pharmacists must follow when providing immunization services from community pharmacies [7].

The requirement for a medical prescription is determined by the authorized dispensing status of the specific pharmaceutical product under Costa Rican regulation rather than by pharmacy-based vaccination as a service itself. Accordingly, the prescription requirement should not be assumed to be identical for all vaccine products. The IQVIA dataset did not contain information on prescription status and therefore does not allow the present analysis to distinguish dispensing according to this characteristic. Costa Rican regulation recognizes different authorized modalities of pharmaceutical sale, including prescription-only and non-prescription products [26].

Yellow fever provides a particularly relevant example of how private-sector vaccination operates in Costa Rica. Yellow fever vaccines obtained in the private sector may be administered in authorized private settings, including pharmacies, clinics, hospitals, and medical or nursing practices. The establishment that administers the vaccine is responsible for recording the vaccination in the national nominal vaccination system (SINOVAC), which supports issuance of the corresponding official vaccination certificate. For international travel purposes, the International Certificate of Vaccination or Prophylaxis becomes valid 10 days after primary yellow fever vaccination and remains valid for the lifetime of the vaccinated person. During the study period, Costa Rica implemented digital certification of yellow fever vaccination, and by 2026 the official certificate was issued digitally [27].

The Costa Rican context can also be considered within the broader international development of pharmacy-based immunization. According to a 2024 report from the International Pharmaceutical Federation (FIP), pharmacy-based vaccination services were available in 56 countries and territories, and pharmacists themselves administered vaccines in 43 jurisdictions. These international data illustrate substantial variation in the regulatory and professional scope of pharmacy-based vaccination and provide context for the Costa Rican model; however, they should not be interpreted as evidence of directly comparable practice across healthcare systems [28].

Despite this regulatory framework, the IQVIA dataset does not identify whether vaccines dispensed during the study period were subsequently administered, whether administration occurred in the dispensing pharmacy or another healthcare setting, who administered the vaccine, or whether a specific pharmacist-led intervention was associated with the dispensed dose. Therefore, the present findings characterize vaccine dispensing through the private community pharmacy channel rather than pharmacist-administered vaccination. Likewise, the dispensing patterns observed in Costa Rica should not be assumed to be directly transferable to countries with different public–private healthcare structures, financing mechanisms, regulatory frameworks, or scopes of pharmacy practice.

### 4.7. Limitations

This study has several limitations inherent to the use of aggregated private-market dispensing data. First, although each unit in the analytical dataset corresponded to one vaccine dose dispensed, the database does not confirm whether the dispensed vaccine was subsequently administered, where administration occurred, who administered it, or the number or characteristics of individuals vaccinated. Consequently, the findings cannot be interpreted as estimates of vaccination coverage, vaccine administration, vaccine uptake, the number of unique individuals vaccinated, or clinical or epidemiological outcomes.

The market coverage of the IQVIA dataset should also be considered when interpreting the findings. The analytical dataset did not provide the proportion of private pharmacies or private vaccine sales captured, nor the underlying sampling, weighting, or projection parameters, preventing independent quantification of private-market coverage. Furthermore, because corresponding public-sector vaccine data were not available, the private-market dispensing estimates cannot be interpreted as representing total vaccine utilization in Costa Rica. The study therefore cannot determine what proportion of national vaccine dispensing or administration occurs through private community pharmacies relative to the CCSS and other public-sector vaccination services.

The absence of information on the underlying sampling design, weighting procedures, projection parameters, and variance estimates also precluded the calculation of statistically valid sampling-based confidence intervals. Consequently, the reported estimates should be interpreted as descriptive measures derived from the available IQVIA dataset rather than as inferential estimates accompanied by quantified sampling uncertainty. The sensitivity analysis excluding yellow fever was performed to assess the influence of the vaccine category with the largest between-period change on the aggregate estimates; however, this analysis does not substitute for statistical measures of sampling uncertainty.

The comparison is based on two consecutive 12-month MAT periods and therefore describes differences between these periods rather than a long-term temporal trend. Although MAT values reduce the influence of isolated monthly fluctuations, the aggregated structure of the data does not permit assessment of monthly or seasonal variation or attribution of observed changes to specific causal factors. Accordingly, contextual epidemiological, regulatory, or market factors discussed in relation to the observed dispensing patterns should be interpreted as temporally associated considerations rather than demonstrated determinants of the changes observed.

## 5. Conclusions

Based on aggregated private-market data, vaccine dispensing in private community pharmacies in Costa Rica was higher in MAT February 2026 than in MAT February 2025, with dispensed doses increasing by 33.6% and economic value by 54.2%. The greater relative increase in economic value reflects differences in the composition of dispensing across vaccine categories. Influenza accounted for the highest dispensing volume, yellow fever showed the largest between-period increase in both dispensed doses and economic value, and HPV maintained substantial economic weight. The sensitivity analysis showed that the magnitude of the aggregate increase was largely concentrated in the yellow fever category, while a more modest positive between-period difference remained after its exclusion. Geographically, dispensing remained concentrated in San José, while several other provinces showed higher dispensing volumes in MAT February 2026 than in MAT February 2025.

Taken together, these findings provide a descriptive characterization of vaccine dispensing through the Costa Rican private community pharmacy market during the two MAT periods analyzed. Although the study does not capture the specific professional interventions associated with each dispensed dose or allow estimation of persons vaccinated, doses administered, vaccination coverage, vaccine uptake, safety, effectiveness, or epidemiological impact, it provides evidence on the volume, economic value, vaccine-category composition, and provincial distribution of this component of the private vaccine market. Because corresponding public-sector data and information on the proportion of the private market captured by the IQVIA dataset were unavailable, the findings should not be interpreted as representing total vaccine utilization in Costa Rica or the proportional contribution of private pharmacies to the national immunization system.

## Figures and Tables

**Figure 1:**
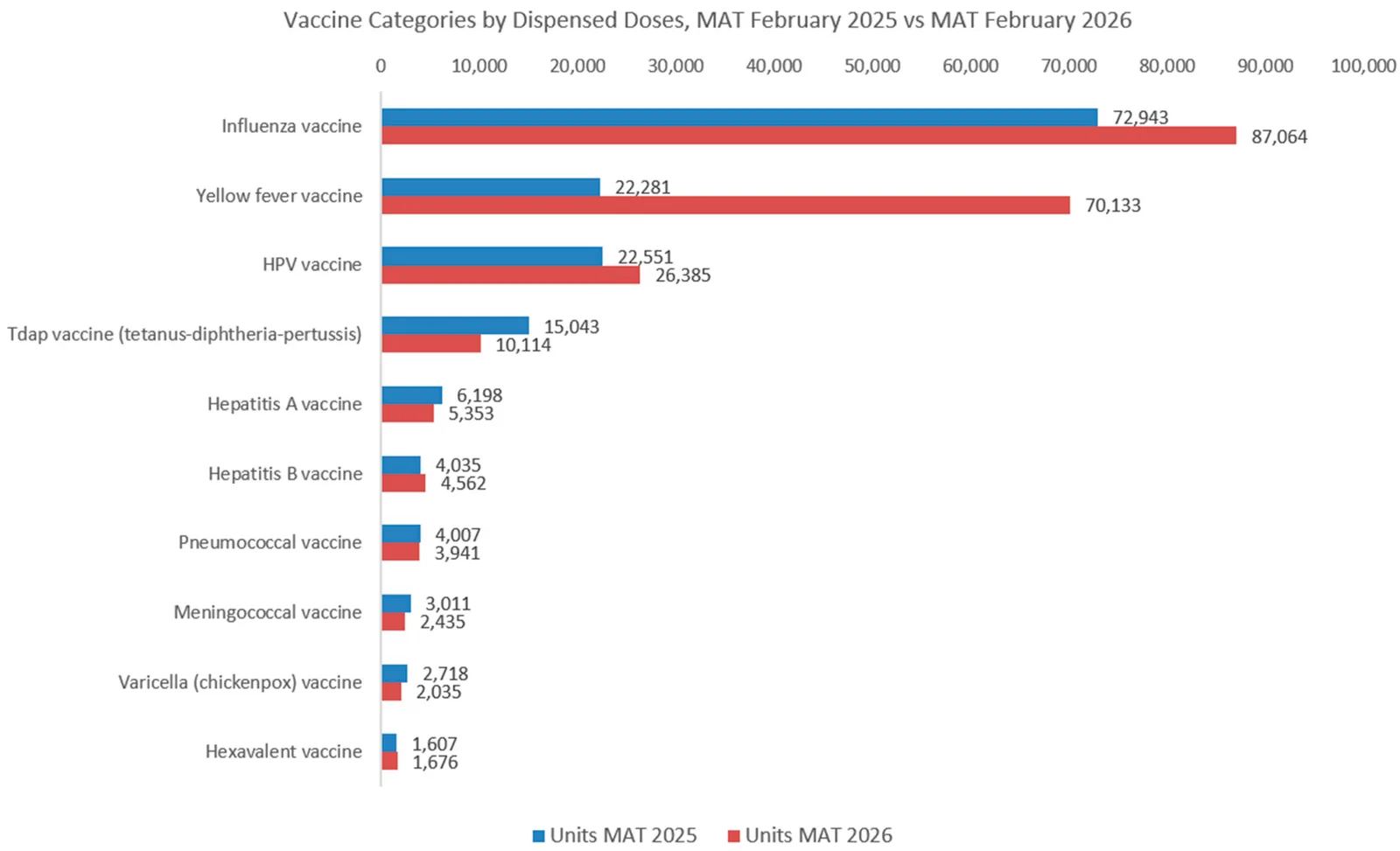
Main vaccine categories by dispensed doses in private community pharmacies in Costa Rica, MAT February 2025 vs. MAT February 2026.

**Figure 2:**
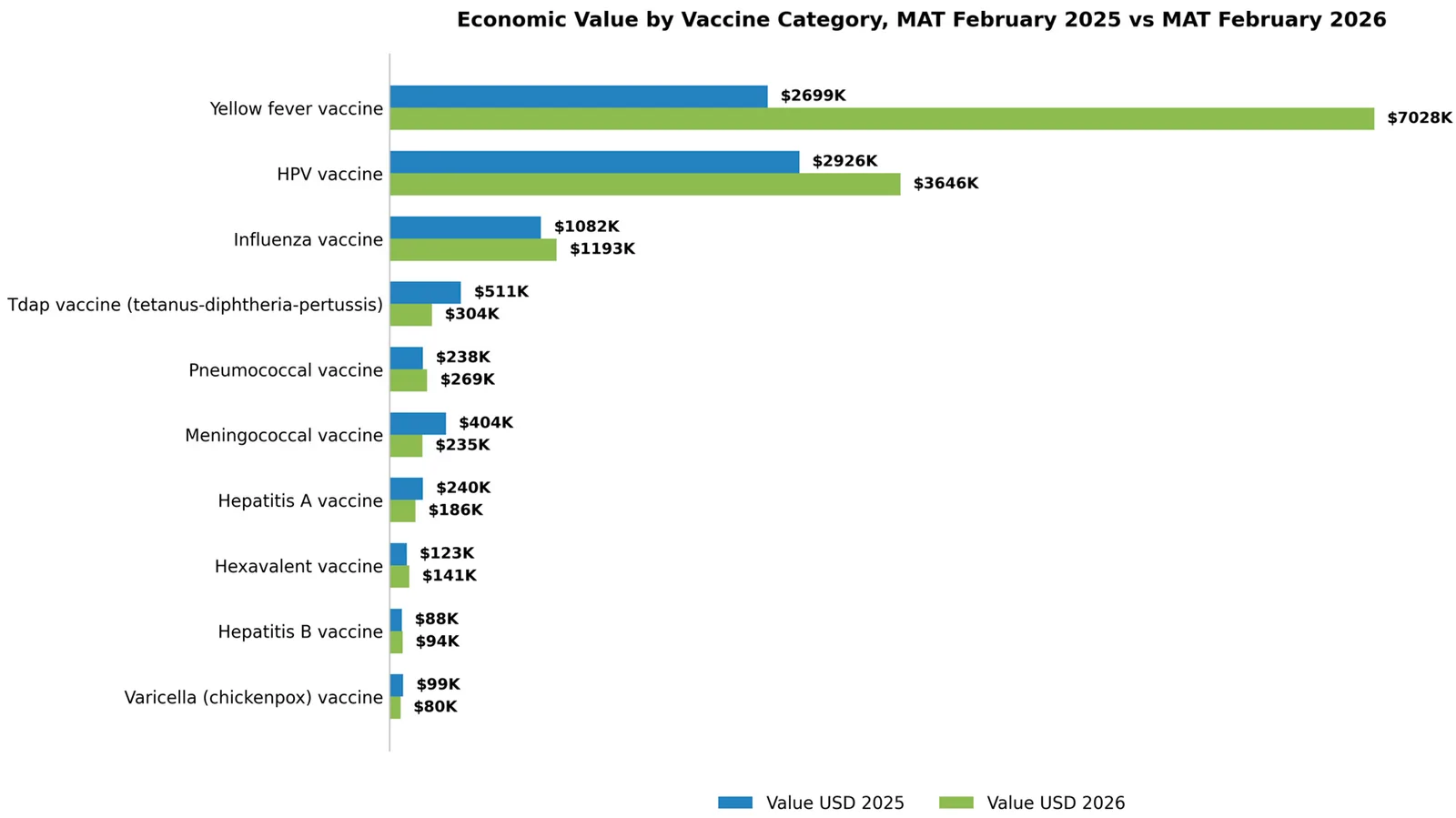
Main vaccine categories by economic value in private pharmacies in Costa Rica, MAT February 2025 vs. MAT February 2026.

**Figure 3:**
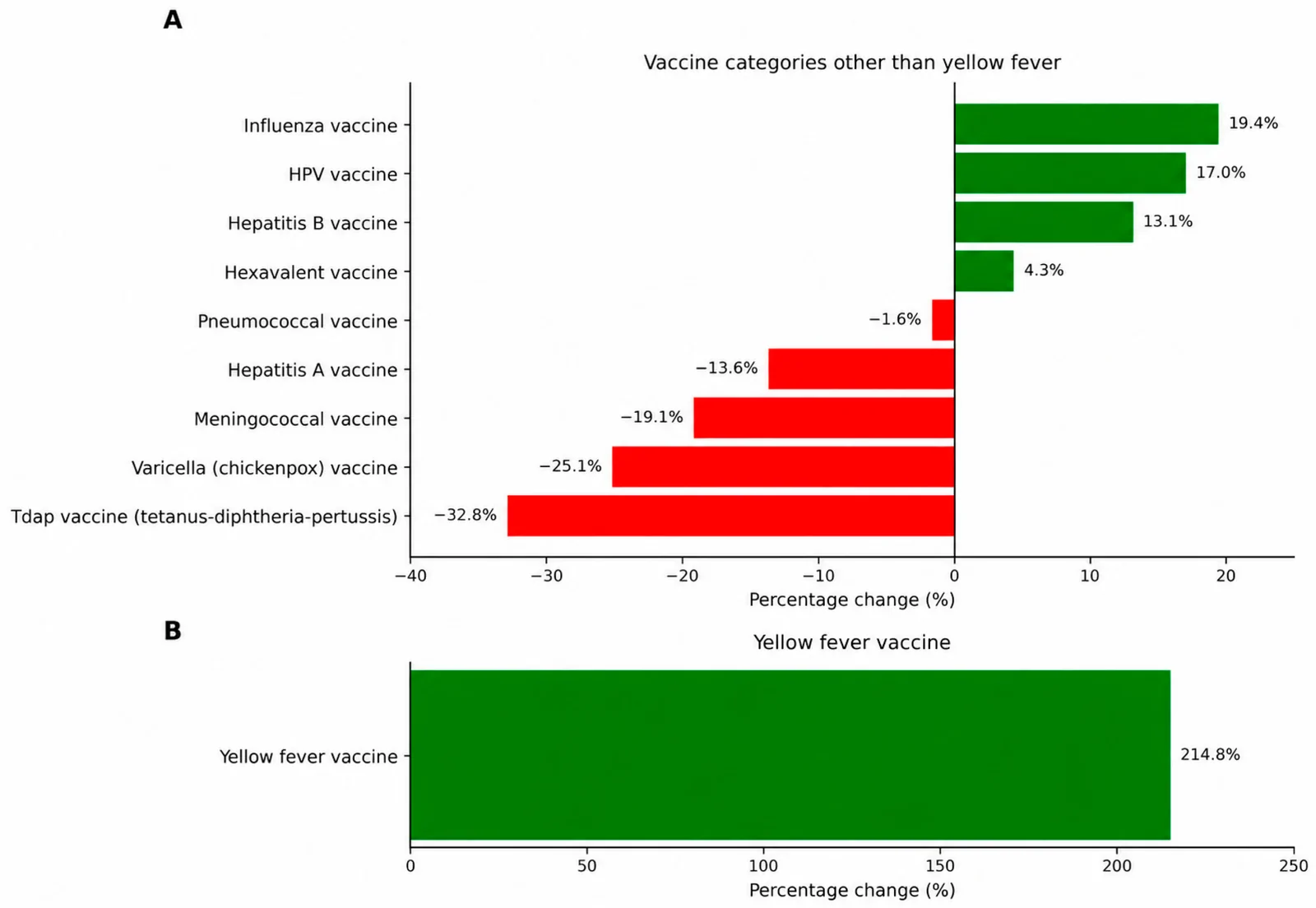
Percentage variation in dispensed doses by vaccine category in private community pharmacies in Costa Rica, MAT February 2025–2026. Panel (**A**) shows the percentage variation for vaccine categories other than yellow fever using an adjusted scale to facilitate comparison. Panel (**B**) displays the percentage variation for yellow fever separately (+214.8%) because its markedly larger increase compressed the scale of the remaining categories in the original graph.

**Figure 4:**
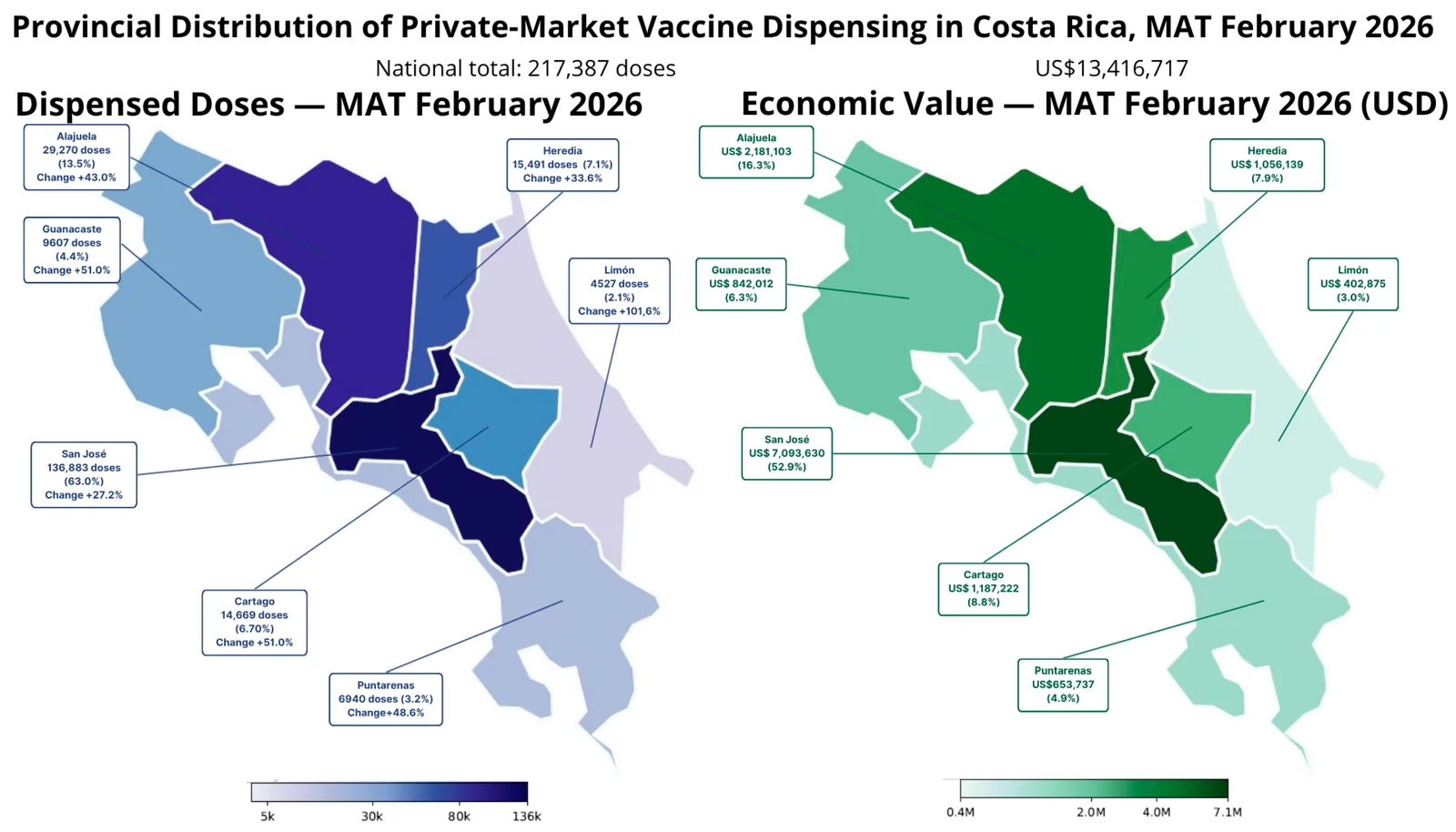
Provincial distribution of dispensed vaccine doses and economic value in private community pharmacies in Costa Rica, MAT February 2026. The left panel shows the number of dispensed doses, the corresponding provincial share of the national dispensing total in parentheses, and the percentage change relative to MAT February 2025. The right panel shows economic value in USD and the corresponding provincial share of the national economic value in parentheses.

**Table 1:** National volume and economic value of preventive vaccines, MAT February 2025–2026.

Indicator	MAT Feb 2025	MAT Feb 2026
Dispensed doses	162,664	217,387
USD value	$8,700,809	$13,416,717

## Data Availability

The data analyzed in this study were obtained from IQVIA under a commercial license and are subject to restrictions; they are not publicly available.

## Supplementary Materials

The following supporting information can be downloaded at: https://www.mdpi.com/article/10.3390/pharmacy14070146/s1: Supplementary Table S1: ATC-based therapeutic classification and analytical vaccine-category mapping used in the study; Supplementary Table S2: Complete vaccine-category distribution in MAT February 2025 and MAT February 2026; Supplementary File S1: STROBE Statement—Checklist of items that should be included in reports of cross-sectional studies.
