# Supplementary material for: Vaccine Dispensing Through Private Community Pharmacies in Costa Rica: A National Descriptive Analysis
Source: Pharmacy (Basel). 2026 Oct 1;14(7):146. doi: 10.3390/pharmacy14070146 (PMC13708262; doi:10.3390/pharmacy14070146)
Supplement: Supplementary file 1 [file pharmacy-14-00146-s001.zip › Supplementary File S1. STROBE Statement.pdf]

STROBE Statement—Checklist of items that should be included in reports of ***cross-sectional studies***

	Item No	Recommendation	Page No
**Title and abstract**	1	(*a*) Indicate the study's design with a commonly used term in the title or the abstract	p. 1 – Title and Abstract
		(*b*) Provide in the abstract an informative and balanced summary of what was done and what was found	p. 1 – Abstract
**Introduction**			
Background/rationale	2	Explain the scientific background and rationale for the investigation being reported	pp. 2–3 – Introduction
Objectives	3	State specific objectives, including any prespecified hypotheses	p. 3 – Introduction, final paragraph (research question and study objective)
**Methods**			
Study design	4	Present key elements of study design early in the paper	p. 3 – Section 2.1, Study Design
Setting	5	Describe the setting, locations, and relevant dates, including periods of recruitment, exposure, follow-up, and data collection	pp. 3–4 – Sections 2.1–2.3, Study Design, Data Source, and Analysis Period
Participants	6	(*a*) Give the eligibility criteria, and the sources and methods of selection of participants	N/A – The study used aggregated secondary private-market data and did not include individual participants. Eligibility criteria for vaccine products are described in p. 5, Sections 2.5–2.6.
Variables	7	Clearly define all outcomes, exposures, predictors, potential confounders, and effect modifiers. Give diagnostic criteria, if applicable	pp. 4–5 – Section 2.7, Variables Analyzed
Data sources/ measurement	8*	For each variable of interest, give sources of data and details of methods of assessment (measurement). Describe comparability of assessment methods if there is more than one group	pp. 3–5 – Sections 2.2, 2.4, and 2.7
Bias	9	Describe any efforts to address potential sources of bias	pp. 3–5 and p. 16 – Data-source coverage limitations, inclusion/exclusion criteria, and Section 4.7, Limitations
Study size	10	Explain how the study size was arrived at	p. 5 – Section 2.5. All eligible preventive vaccine products available in the IQVIA analytical dataset were included; no sample-size calculation was performed.

Quantitative variables	11	Explain how quantitative variables were handled in the analyses. If applicable, describe which groupings were chosen and why	pp. 5–7 – Sections 2.7–2.8, Variables Analyzed and Data Analysis
Statistical methods	12	(*a*) Describe all statistical methods, including those used to control for confounding	pp. 6–7 – Section 2.8, Data Analysis. Descriptive analyses only; no inferential models or confounder adjustment were performed.
		(*b*) Describe any methods used to examine subgroups and interactions	N/A – No inferential subgroup or interaction analyses were performed. Descriptive results were stratified by vaccine category and province.
		(*c*) Explain how missing data were addressed	p. 5 – Section 2.6, Exclusion Criteria. Empty rows, totals, subtotals, and duplicate records were excluded during data cleaning.
		(*d*) If applicable, describe analytical methods taking account of sampling strategy	pp. 3 and 5 – The available dataset did not provide the sampling frame, sampling weights, projection parameters, or variance estimates; therefore, sampling-based inference was not performed.
		(*e*) Describe any sensitivity analyses	pp. 6–7 – Section 2.8; p. 10 – Results. Sensitivity analysis excluding yellow fever vaccine.
**Results**			
Participants	13*	(a) Report numbers of individuals at each stage of study—eg numbers potentially eligible, examined for eligibility, confirmed eligible, included in the study, completing follow-up, and analysed	N/A – No individual participants were enrolled; the analysis used aggregated private-market vaccine records.
		(b) Give reasons for non-participation at each stage	N/A – No individual participant recruitment or participation occurred.
		(c) Consider use of a flow diagram	N/A – No participant recruitment or follow-up flow was applicable.
Descriptive data	14*	(a) Give characteristics of study participants (eg demographic, clinical, social) and information on exposures and potential confounders	N/A for individual characteristics – No patient-level demographic or clinical data were available. Aggregate vaccine dispensing characteristics are reported on pp. 7–11.

		(b) Indicate number of participants with missing data for each variable of interest	N/A – No individual-level participant data were analyzed.
Outcome data	15*	Report numbers of outcome events or summary measures	pp. 7–11 – Results, Table 1, and Figures 1–4
Main results	16	(*a*) Give unadjusted estimates and, if applicable, confounder-adjusted estimates and their precision (eg, 95% confidence interval). Make clear which confounders were adjusted for and why they were included	pp. 7–11 – Results. Descriptive unadjusted estimates are reported; adjusted estimates and sampling-based confidence intervals were not applicable given the available aggregated dataset.
		(*b*) Report category boundaries when continuous variables were categorized	N/A – Continuous variables were not categorized into analytical groups.
		(*c*) If relevant, consider translating estimates of relative risk into absolute risk for a meaningful time period	N/A – No relative-risk estimates were calculated.
Other analyses	17	Report other analyses done—eg analyses of subgroups and interactions, and sensitivity analyses	p. 10 – Sensitivity analysis excluding yellow fever vaccine
**Discussion**			
Key results	18	Summarise key results with reference to study objectives	pp. 11–16 – Discussion, Sections 4.1–4.6
Limitations	19	Discuss limitations of the study, taking into account sources of potential bias or imprecision. Discuss both direction and magnitude of any potential bias	p. 16 – Section 4.7, Limitations
Interpretation	20	Give a cautious overall interpretation of results considering objectives, limitations, multiplicity of analyses, results from similar studies, and other relevant evidence	pp. 11–16 – Discussion
Generalisability	21	Discuss the generalisability (external validity) of the study results	pp. 15–17 – Sections 4.6–4.7 and Conclusions
**Other information**			
Funding	22	Give the source of funding and the role of the funders for the present study and, if applicable, for the original study on which the present article is based	p. 17 – Funding Statement

*Give information separately for exposed and unexposed groups.

Note: An Explanation and Elaboration article discusses each checklist item and gives methodological background and published examples of transparent reporting. The STROBE checklist is best used in conjunction with this article (freely available on the Web sites of PLoS Medicine at http://www.plosmedicine.org/, Annals of Internal Medicine at http://www.annals.org/, and Epidemiology at http://www.epidem.com/). Information on the STROBE Initiative is available at www.strobe-statement.org.
