# Supplementary material for: Vaccine Dispensing Through Private Community Pharmacies in Costa Rica: A National Descriptive Analysis
Source: Pharmacy (Basel). 2026 Oct 1;14(7):146. doi: 10.3390/pharmacy14070146 (PMC13708262; doi:10.3390/pharmacy14070146)
Supplement: Supplementary file 1 [file pharmacy-14-00146-s001.zip › Supplementary Table S1.pdf]

ATC-based therapeutic subgroup in the source dataset	Source therapeutic subgroup	Analytical vaccine category	Classification approach
J07B1	Combinations with tetanus	Tdap vaccine	Combination vaccine retained as a distinct analytical category according to antigen composition
J07B1	Combinations with tetanus	DTaP-polio vaccine	Combination vaccine retained as a distinct analytical category according to antigen composition
J07B1	Combinations with tetanus	Hexavalent vaccine	Combination vaccine retained as a distinct analytical category according to antigen composition
J07B2	Combinations with measles and/or mumps	MMR vaccine	Combination vaccine retained as a distinct analytical category according to antigen composition
J07D1	Pneumococcal vaccines	Pneumococcal vaccine	Eligible records aggregated within the corresponding vaccine category
J07D2	Meningococcal vaccines	Meningococcal vaccine	Eligible records aggregated within the corresponding vaccine category
J07D4	Typhoid vaccines	Typhoid vaccine	Eligible records aggregated within the corresponding vaccine category
J07E1	Influenza vaccines	Influenza vaccine	Eligible records aggregated within the corresponding vaccine category
J07E2	Varicella-zoster vaccines	Varicella (chickenpox) vaccine	Assigned according to vaccine antigen/composition

ATC-based therapeutic subgroup in the source dataset	Source therapeutic subgroup	Analytical vaccine category	Classification approach
J07E2	Varicella-zoster vaccines	Shingles (herpes zoster) vaccine	Assigned according to vaccine antigen/composition
J07E3	Human papillomavirus vaccines	HPV vaccine	Eligible records aggregated within the corresponding vaccine category
J07E4	Hepatitis vaccines	Hepatitis A vaccine	Assigned according to vaccine antigen/composition
J07E4	Hepatitis vaccines	Hepatitis B vaccine	Assigned according to vaccine antigen/composition
J07E5	Rotavirus vaccines	Rotavirus vaccine	Eligible records aggregated within the corresponding vaccine category
J07E7	Respiratory syncytial virus vaccines	RSV vaccine	Eligible records aggregated within the corresponding vaccine category
J07E9	Other viral vaccines	Yellow fever vaccine	Assigned according to vaccine antigen/composition
J07E9	Other viral vaccines	Rabies vaccine	Assigned according to vaccine antigen/composition

Note: Therapeutic subgroup codes correspond to the ATC-based classification provided in the IQVIA source dataset. Because some therapeutic subgroups encompassed more than one vaccine type, analytical category assignment was further determined according to vaccine antigen composition. Each eligible product record was assigned to one mutually exclusive analytical category. Combination vaccines were retained as distinct categories and were not apportioned among their individual antigen components, thereby avoiding double counting. Commercial product names, manufacturers, presentations, and source-level product identifiers are not reproduced because the underlying IQVIA dataset was obtained under a commercial license and is not publicly available.
