# Supplementary material for: Vaccine Dispensing Through Private Community Pharmacies in Costa Rica: A National Descriptive Analysis
Source: Pharmacy (Basel). 2026 Oct 1;14(7):146. doi: 10.3390/pharmacy14070146 (PMC13708262; doi:10.3390/pharmacy14070146)
Supplement: Supplementary file 1 [file pharmacy-14-00146-s001.zip › Supplementary Table S2.pdf]

Vaccine category	Doses 2025	Dose share 2025 (%)	Doses 2026	Dose share 2026 (%)	Change in doses (%)	Economic value 2025 (USD)	Value share 2025 (%)	Economic value 2026 (USD)	Value share 2026 (%)	Change in value (%)	Value/dose 2025 (USD)	Value/dose 2026 (USD)
Influenza vaccine	72,943	44.8	87,064	40.1	+19.4	1,082,446.91	12.4	1,193,490.29	8.9	+10.3	14.84	13.71
Yellow fever vaccine	22,281	13.7	70,133	32.3	+214.8	2,698,642.52	31.0	7,027,781.76	52.4	+160.4	121.12	100.21
HPV vaccine	22,551	13.9	26,385	12.1	+17.0	2,925,908.78	33.6	3,646,239.90	27.2	+24.6	129.75	138.19
Tdap vaccine (tetanus-diphtheria-pertussis)	15,043	9.2	10,114	4.7	−32.8	511,475.69	5.9	303,636.23	2.3	−40.6	34.00	30.02
Hepatitis A vaccine	6,198	3.8	5,353	2.5	−13.6	239,710.76	2.8	186,210.97	1.4	−22.3	38.68	34.79
Hepatitis B vaccine	4,035	2.5	4,562	2.1	+13.1	87,620.31	1.0	93,714.96	0.7	+7.0	21.72	20.54
Pneumococcal vaccine	4,007	2.5	3,941	1.8	−1.6	238,079.09	2.7	269,447.16	2.0	+13.2	59.42	68.37

Vaccine category	Doses 2025	Dose share 2025 (%)	Doses 2026	Dose share 2026 (%)	Change in doses (%)	Economic value 2025 (USD)	Value share 2025 (%)	Economic value 2026 (USD)	Value share 2026 (%)	Change in value (%)	Value/dose 2025 (USD)	Value/dose 2026 (USD)
Meningococcal vaccine	3,011	1.9	2,435	1.1	−19.1	404,053.25	4.6	235,498.89	1.8	−41.7	134.19	96.71
Varicella (chickenpox) vaccine	2,718	1.7	2,035	0.9	−25.1	98,634.40	1.1	79,641.57	0.6	−19.3	36.29	39.14
Hexavalent vaccine	1,607	1.0	1,676	0.8	+4.3	123,067.08	1.4	140,781.10	1.0	+14.4	76.58	84.00
MMR vaccine (measles-mumps-rubella)	6,353	3.9	1,540	0.7	−75.8	172,765.05	2.0	46,459.18	0.3	−73.1	27.19	30.17
Rotavirus vaccine	972	0.6	896	0.4	−7.8	79,053.96	0.9	71,680.92	0.5	−9.3	81.33	80.00
DTaP-polio vaccine	383	0.2	400	0.2	+4.4	12,667.53	0.1	10,465.55	0.1	−17.4	33.07	26.16

Vaccine category	Doses 2025	Dose share 2025 (%)	Doses 2026	Dose share 2026 (%)	Change in doses (%)	Economic value 2025 (USD)	Value share 2025 (%)	Economic value 2026 (USD)	Value share 2026 (%)	Change in value (%)	Value/dose 2025 (USD)	Value/dose 2026 (USD)
Shingles (herpes zoster) vaccine	1	<0.1	378	0.2	+37,700.0	335.01	<0.1	62,878.55	0.5	+18,669.2	335.01	166.35
Rabies vaccine	440	0.3	311	0.1	−29.3	21,679.94	0.2	12,694.56	0.1	−41.4	49.27	40.82
RSV vaccine	0	0.0	162	0.1	N/A	0.00	0.0	36,023.27	0.3	N/A	N/A	222.37
Typhoid vaccine	121	0.1	2	<0.1	−98.3	4,668.31	0.1	72.34	<0.1	−98.5	38.58	36.17
**Total**	**162,664**	**100.0**	**217,387**	**100.0**	**+33.6**	**8,700,808.59**	**100.0**	**13,416,717.20**	**100.0**	**+54.2**	**53.49**	**61.72**

Abbreviations: HPV, human papillomavirus; Tdap, tetanus-diphtheria-acellular pertussis; DTaP, diphtheria-tetanus-acellular pertussis; MMR, measles-mumps-rubella; RSV, respiratory syncytial virus; MAT, moving annual total. Percentage change was not calculated when the MAT February 2025 value was zero. Average economic value per dispensed dose was calculated as aggregate economic value divided by the corresponding number of dispensed doses. Values may not sum exactly to 100.0% because of rounding.
